# P-2152. Adenovirus Viremia in Adults: Clinical Syndromes, Risk Factors, and Outcomes

**DOI:** 10.1093/ofid/ofaf695.2315

**Published:** 2026-01-11

**Authors:** Abdallah Mughrabi, Douglas Challener, Madiha Fida, Raymund R Razonable, Omar M Abu Saleh

**Affiliations:** Mayo Clinic, Rochester, Minnesota; Mayo Clinic, Rochester, Minnesota; Mayo Clinic, Rochester, Minnesota; Mayo Clinic, Rochester, Minnesota; Mayo Clinic, Rochester, Minnesota

## Abstract

**Background:**

Adenovirus is a ubiquitous dsDNA virus with potential for end-organ disease. End-organ or disseminated adenoviral disease (EOD) occurs more frequently in hematopoietic stem cell transplant (HSCT) and solid organ transplant (SOT) recipients. We aim to characterize the clinical manifestations and outcome of adenovirus viremia in adults, including HSCT and SOT recipients. We describe severity of illness, organ-involvement, and graft and patient survival.

**Figure:**
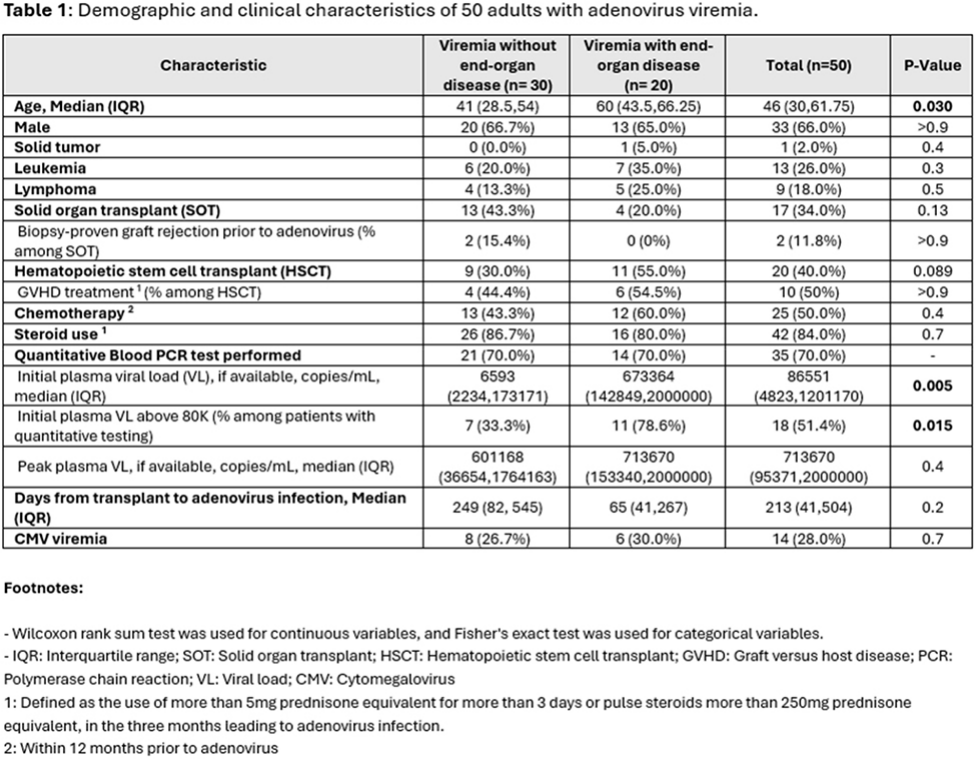

**Figure:**
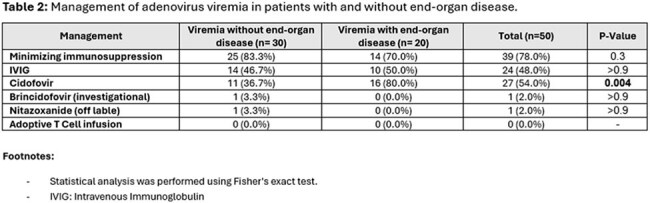

**Methods:**

This retrospective, multi-site, study enrolled adults patients with positive adenovirus PCR in blood and other clinical specimens from January, 2010 through April, 2025. We excluded patients with insufficient medical records, follow up less than 30 days, and isolated conjunctivitis without viremia. EOD was defined by the presence of clinical signs or symptoms consistent with organ involvement, confirmed by a positive adenovirus PCR or by histopathology. Descriptive and comparative statistics were performed using R.

**Figure:**
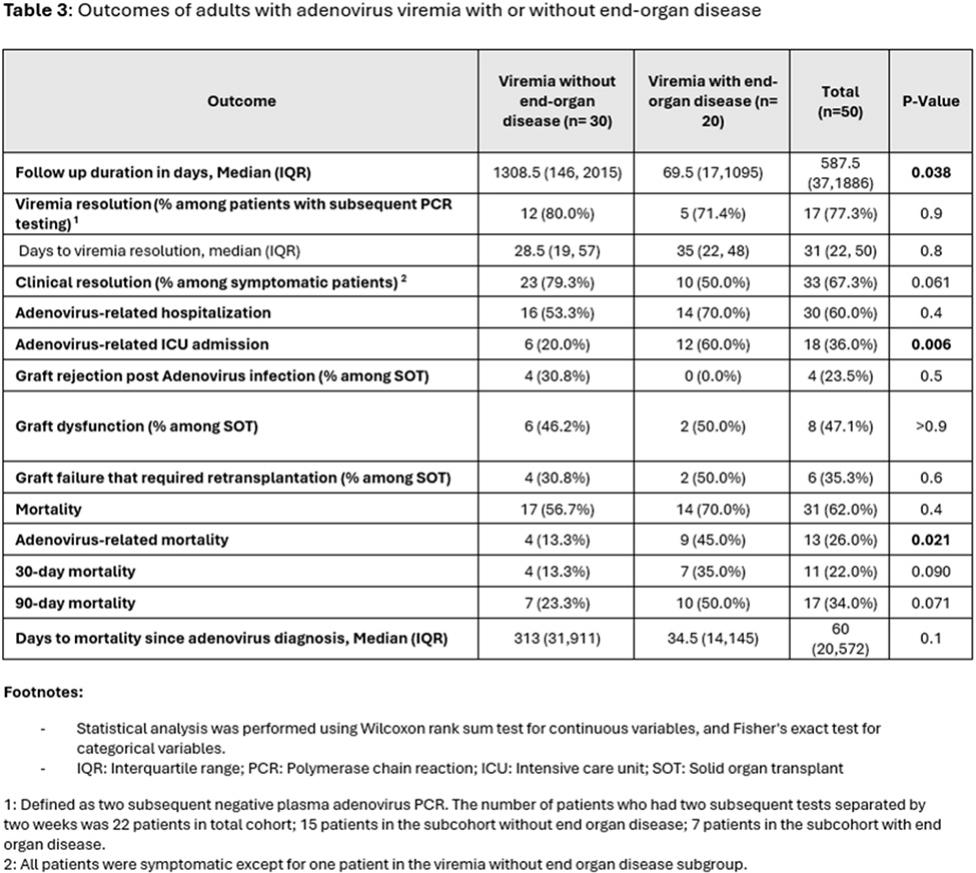

**Figure:**
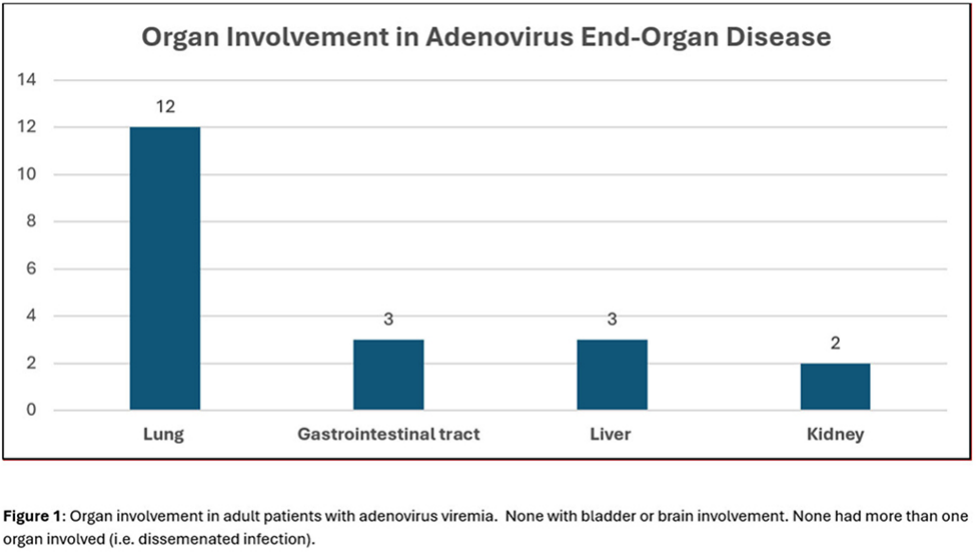

**Results:**

Fifty patients with adenovirus viremia were included in this study. The most common immunocompromising conditions were corticosteroid use (84%, n = 42), HSCT (40%, n = 20) and SOT (34%, n = 17) (Table 1). Only 3 patients (6%) had no known immunocompromising condition. 20 patients (40%) had EOD while 30 patients (60%) had isolated viremia. Lungs were the most affected organ, followed by the gastrointestinal tract and liver (Figure 1). EOD was more prevalent in older patients (60 vs. 41 years, p =0.03) and in those with higher initial plasma viral load (673364 vs. 6593, p=0.005). The most common treatment was reduction in immunosuppression (Table 2). Cidofovir was more often used in patients with EOD. Adenovirus-related outcomes were worse in patients with EOD [higher rates of mortality (45% vs. 13.3%) and ICU admission (60% vs. 20%)] vs. without EOD (Table 3).

**Conclusion:**

Adenovirus viremia is associated with high mortality in a cohort of mostly immunocompromised adults, particularly those with EOD. Older age and a higher initial plasma viral load are associated with EOD. Patients with EOD had worse clinical outcomes such as ICU admission and adenovirus-related mortality.

**Disclosures:**

All Authors: No reported disclosures

